# Baseline serum 25 hydroxyvitamin D concentration does not predict functional recovery in intensive inpatient rehabilitation: a retrospective observational study

**DOI:** 10.3389/fnut.2026.1966784

**Published:** 2026-10-08

**Authors:** Federica Bellone, Angelo Alito, Laura Turriziani, Giuseppe Spadaro, Simone Crescimone, Antonino Catalano, Eliana Cipolla, Fabio Malacarne, Giovanni Squadrito, Giorgio Basile

**Affiliations:** 1 Department of Clinical and Experimental Medicine, University of Messina, Messina, Italy; 2 Brain Mapping Lab, Department of Biomedical, Dental Sciences and Morphological and Functional Imaging, University of Messina, Messina, Italy; 3 IRCCS Centro Neurolesi Bonino-Pulejo, Messina, Italy; 4 Geriatric Unit, University Hospital "G. Martino", Messina, Italy; 5 Department of Biomedical, Dental Sciences and Morphological and Functional Imaging, University of Messina, Messina, Italy

**Keywords:** 25-hydroxyvitamin D, Barthel Index, Functional Independence Measure [FIM(SM) instrument], functional outcome, null results, rehabilitation, vitamin D

## Abstract

**Introduction:**

Vitamin D deficiency is highly prevalent among hospitalized patients, and its effects on skeletal muscle function and systemic inflammation suggest that baseline status might influence functional recovery; however, evidence specifically in intensive inpatient rehabilitation remains scarce.

**Methods:**

In this retrospective observational cohort study, 97 adults (mean age 65.0 ± 13.1 years) admitted to intensive inpatient rehabilitation were enrolled. Serum 25-hydroxyvitamin D [25(OH)D] obtained within 48 h of admission was analyzed both as a continuous variable and categorized into deficient, insufficient, and sufficient groups. The primary outcome was the Functional Independence Measure (FIM) at discharge adjusted for admission score; secondary outcomes included the Barthel Index (BI), Functional Ambulation Category (FAC), 6-min walk test (6MWT), and length of stay. Hierarchical linear regression was performed adjusting for admission functional status, age, comorbidity burden, and diagnostic group, with robust standard errors and bootstrap confidence intervals.

**Results:**

Hypovitaminosis D affected 78.4% of patients. Despite substantial functional improvement across the cohort, baseline serum 25(OH)D was not associated with any outcome measure—neither as a continuous variable nor across vitamin D categories—in unadjusted or fully adjusted analyses. Admission functional status (i.e., FIM) was the dominant predictor in all models.

**Discussion:**

Baseline serum 25(OH)D did not predict functional recovery during intensive inpatient rehabilitation, irrespective of outcome measure. Although hypovitaminosis D was highly prevalent and warrants correction for skeletal health, the present study did not find evidence to support the use of serum 25(OH)D as a prognostic marker of rehabilitation outcome.

## 1 Introduction

Vitamin D is a pleiotropic hormone with crucial roles beyond bone metabolism, including neuromuscular function, immune regulation, and inflammatory control (1–3). Vitamin D receptors (VDR) are widely expressed not only in bone and intestinal tissue but also in skeletal muscle, immune cells, and the central nervous system, underscoring the hormone’s broad physiological relevance (4). Particularly, in skeletal muscle, vitamin D signaling influences myocyte differentiation, calcium handling, and protein synthesis, and its deficiency has been consistently associated with reduced muscle strength, impaired physical performance, and increased risk of falls in older adults (5, 6). This mechanistic link is particularly relevant in the context of sarcopenia, a progressive condition characterized by loss of muscle mass and function that shares several risk factors and pathophysiological pathways with hypovitaminosis D (7, 8).

Beyond the musculoskeletal system, vitamin D exerts immunomodulatory effects by regulating both innate and adaptive immune responses, promoting antimicrobial peptide production while reducing excessive pro-inflammatory cytokine release (9, 10). Low serum 25-hydroxyvitamin D [25(OH)D] levels have therefore been implicated in a wide range of conditions, including respiratory infections, autoimmune diseases, and chronic low-grade inflammation, a hallmark of aging often referred to as “inflammaging” (11). Older adults are particularly vulnerable to vitamin D deficiency due to a combination of factors, including reduced cutaneous synthesis related to skin aging, limited sun exposure, decreased dietary intake, impaired intestinal absorption, and age-related decline in renal hydroxylation capacity (12–14). This vulnerability is further amplified in hospitalized or institutionalized populations. Reduced mobility and comorbidities compound the risk in these settings, contributing to the high prevalence rates reported (15–17).

Patients admitted to intensive inpatient rehabilitation facilities represent a population at particularly high risk of vitamin D insufficiency, owing to prolonged immobilization, reduced sun exposure, acute-phase inflammatory responses, and the frailty and comorbidity burden typical of this setting (17, 18). In rehabilitation medicine, vitamin D status has been hypothesized to influence recovery trajectories. The proposed mechanisms include improved neuromuscular performance through enhanced calcium signaling and muscle protein synthesis (19–21); modulation of systemic inflammation, potentially accelerating neural plasticity post-stroke or traumatic injury (22, 23); and enhanced immune function, reducing infection-related complications that frequently impair rehabilitation progress (24, 25). Despite these biologically plausible mechanisms, prospective evidence directly linking baseline vitamin D status to functional rehabilitation outcomes remains limited, particularly in mixed orthopedic-neurological populations undergoing intensive inpatient rehabilitation (26, 27).

Previous observational studies in traumatic brain injury (28) and chronic spinal cord injury (29) reported associations between low vitamin D and worse outcomes; however, interventional trials have yielded inconsistent results. Recent randomized controlled trials (RCTs) in post-stroke rehabilitation (22) and severe acquired brain injury (30) found no significant benefit of vitamin D supplementation on functional recovery despite successful elevation of serum levels, suggesting that baseline status alone may not predict outcome independent of intervention.

The International Classification of Functioning, Disability and Health (ICF) framework (31) offers a complementary description of rehabilitation status, capturing body functions such as muscle power (b730) and activities such as walking (d450).

To our knowledge, no study has systematically evaluated baseline vitamin D status as a predictor of functional outcomes across multiple domains in a mixed orthopaedic–neurological intensive rehabilitation cohort. This retrospective observational study investigates whether baseline serum 25(OH)D concentration is associated with functional recovery. The study considers 25(OH)D concentration as both a continuous predictor and a categorical variable defined by established clinical thresholds. Particular attention is given to whether deficient levels predict poorer outcomes compared with sufficient levels.

## 2 Materials and methods

### 2.1 Study design and setting

This was a retrospective observational cohort study based on routinely collected clinical data conducted at the Department of Physical Medicine and Rehabilitation, University Hospital G. Martino (Messina, Italy). Data were collected from consecutive patients admitted to intensive inpatient rehabilitation (providing a multidisciplinary program of 180 min per day, delivered in two sessions per day, 6 days per week) between January 2024 and September 2025, at both admission and discharge. Ethical approval was obtained from the Local Ethics Committee of Messina (approval number: 44–25, approved on 11 November 2025) and authorized by the hospital administration. Reporting follows the Strengthening the Reporting of Observational Studies in Epidemiology (STROBE) guidelines (32). All patients received an intensive, standardized, multidisciplinary rehabilitation program. This was delivered by a team of physiatrists, physiotherapists and speech and language therapists (when clinically indicated). The program encompassed motor rehabilitation, functional training for activities of daily living and gait rehabilitation. The daily dose was uniform across diagnostic groups.

### 2.2 Study population

Inclusion criteria were: (1) age ≥18 years; (2) ordinary hospital admission for intensive rehabilitation; (3) serum vitamin D measurement within 48 h of admission; (4) completion of the functional assessment battery at both admission and discharge; (5) length of stay ≥5 days. Exclusion criteria were: (1) chronic treatment with active vitamin D metabolites (calcitriol, calcifediol, 1-alpha-calcidiol) before admission, as these agents elevate 1,25-dihydroxyvitamin D independently of serum 25(OH)D and would confound the primary exposure; (2) terminal illness or severe cognitive impairment precluding reliable functional assessment; (3) missing vitamin D measurement. Of 118 patients screened, 97 met all criteria (Figure 1). Vitamin D supplementation during admission followed standard clinical practice: patients with deficient 25(OH)D levels were started on cholecalciferol; those already receiving cholecalciferol before admission continued their pre-existing regimen; and those with sufficient levels received no supplementation. Supplementation was recorded and a sensitivity analysis restricted to the 64 non-supplemented patients was performed.

**Figure 1:**
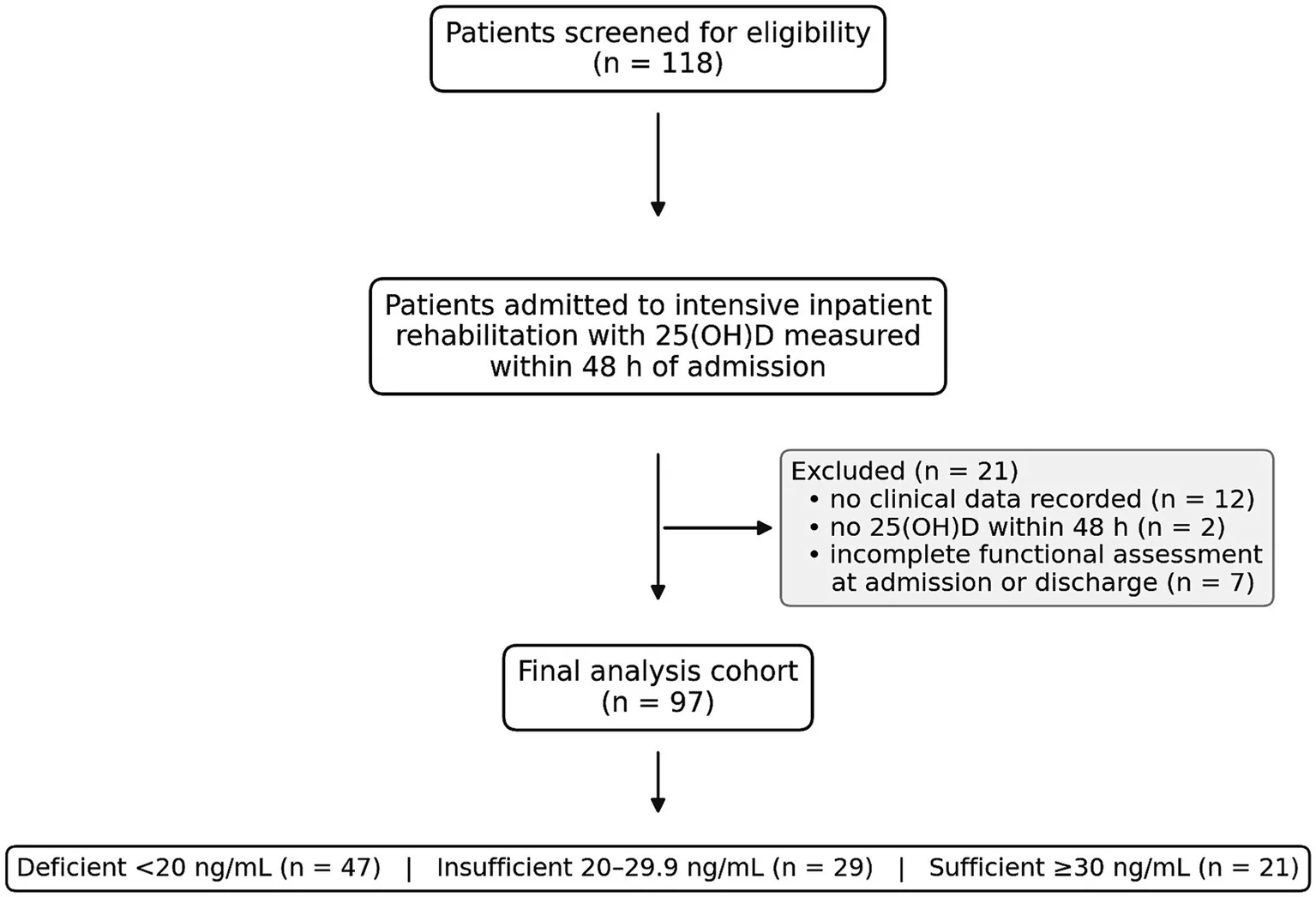
Study flow diagram.

### 2.3 Data collection and variables

Demographic and clinical data were extracted from medical records. Vitamin D status was assessed as serum 25(OH)D concentration and was quantified by High-Performance Liquid Chromatography (HPLC) at the institutional laboratory. The SIOMMMS clinical thresholds (33) were applied for categorical classification: deficient (<20 ng/mL), insufficient (20–29.9 ng/mL) and sufficient (≥30 ng/mL) (33).

Additional variables were age, sex, primary diagnosis, diagnostic group (orthopedic versus neurological), comorbidity burden on the Cumulative Illness Rating Scale (CIRS) (34);the laboratory parameters C-reactive protein, hemoglobin and serum calcium. Functional status was assessed with the Barthel Index (BI) (0–100) (35), the total Functional Independence Measure (FIM, 13–126) (36), the Functional Ambulation Category (FAC, 0–5) (37), and the distance covered in the 6-min walk test (6MWT) (38) (6MWT, meters). ICF (31) qualifiers for muscle power (b730) and walking (d450) were recorded at admission. Changes were computed as discharge value minus admission value, with positive values indicating improvement.

### 2.4 Outcomes

The primary outcome was the total FIM score at discharge, adjusted for the admission FIM score using a regression model. The secondary outcomes were the BI, FAC, 6MWT distance, change in FIM and Bl, and length of stay at discharge. Since all patients recorded 0 m on the 6MWT at admission, reflecting inability to complete the test regardless of residual ambulatory capacity, the 6MWT distance at discharge also represents the change from baseline.

In line with standard practice in intensive inpatient rehabilitation, the total FIM score was used as the primary outcome. Since orthopedic patients undergoing intensive rehabilitation must be fully aware and cooperative, the cognitive subscale tends to be high in this group. However, it may vary in neurological patients due to language and comprehension deficits. ICF qualifiers were recorded at admission only and reported as baseline descriptors of impairment, not outcomes.

### 2.5 Statistical analysis

Descriptive statistics (mean ± standard deviation, median, interquartile range, range, and frequencies) characterized the cohort overall and by vitamin D category. Normality was assessed using the Shapiro–Wilk test, as well as by inspecting histograms and Q–Q plots. Discharge scores departed markedly from normality due to a ceiling effect (Shapiro–Wilk W = 0.66, *p* < 0.001 for FIM at discharge; 44.3% of patients achieved the maximum Barthel score). The variance of FIM at discharge was five times greater among neurological patients than orthopedic patients. Therefore, parametric estimates are reported alongside nonparametric and robust equivalents. Univariate associations between serum 25(OH)D and outcomes were quantified by Pearson correlation coefficients, with Spearman rank coefficients reported in parallel. One-way ANOVA was used to assess differences across the three vitamin D categories for continuous variables, and the chi-square test was used for categorical variables. FAC was treated as a continuous variable, consistent with common practice in rehabilitation research for six-category ordinal scales with approximately equal clinical intervals; the primary inferential findings relied on regression models with HC3 standard errors and bootstrap confidence intervals, which impose no distributional assumptions.

Hierarchical multivariable linear regression was used to test 25(OH)D as a predictor of the primary outcome. Model 1 included 25(OH)D and FIM at admission. Model 2 included age, CIRS, and diagnostic group. Model 3 included C-reactive protein and serum calcium.

Covariates were selected *a priori* based on established clinical and biological rationale: age and CIRS capture the main determinants of functional capacity independent of diagnosis; diagnostic group accounts for fundamentally different recovery mechanisms between orthopedic and neurological patients; CRP reflects systemic inflammation as a potential mediator of the vitamin D–muscle relationship; and serum calcium is a direct metabolic consequence of vitamin D status. Sex was additionally included in the fully adjusted model.

All models were estimated using the entire cohort (*n* = 97) because no covariate had missing values. The regression coefficients (*β*), standard errors, *t* statistics, 95% confidence intervals, and *p*-values are reported. Due to the non-normality of the residuals and between-group heteroscedasticity, the fully adjusted model was re-estimated using heteroscedasticity-consistent standard errors. A 95% confidence interval for the 25(OH)D coefficient was derived from 5,000 bootstrap resamples.

We assessed multicollinearity using variance inflation factors, considering values below 5 to be acceptable. Secondary outcomes were modeled with the same covariate structure as Model 2. Since each model adjusts for the corresponding admission score, the estimates for the discharge value and the change score are algebraically identical; only the former is reported. Sensitivity analyses examined patients with below-median FIM scores at admission, stratification by diagnostic group, and a 25(OH)D × diagnostic group interaction term. With 97 patients and eight covariates, the study had 80% power to detect a partial correlation of 0.285 between 25(OH)D and the primary outcome, corresponding to an incremental *R*^2^ of 0.081. Thus, the analysis was adequately powered to detect moderate effects but not small ones. Statistical significance was set at *α* = 0.05 (two-tailed). Analyses were performed using Jamovi version 2.4.8 (The Jamovi Project, Sydney, Australia).

## 3 Results

### 3.1 Participant characteristics and vitamin D distribution

Of the 118 patients who were screened, 97 met the inclusion criteria (see Figure 1). The mean age was 65.0 ± 13.1 years (range 22–85), and 52 patients (53.6%) were female. The orthopedic group (*n* = 61, 62.9%) comprised patients following total hip arthroplasty (*n* = 30), total knee arthroplasty (*n* = 11), post-femur fracture rehabilitation (*n* = 16), and other trauma undergoing orthopedic surgery (*n* = 4). The neurological group (*n* = 36, 37.1%) comprised predominantly post-stroke patients (*n* = 27), with a minority of Guillain–Barré syndrome (*n* = 2), critical illness myopathy/neuropathy (*n* = 2), post-neurosurgical cases (*n* = 4), and multiple sclerosis (*n* = 1). The mean comorbidity burden was 3.89 ± 1.71 on the CIRS. The median length of stay was 31 days [interquartile range (IQR) 28–38; range 14–81; mean 36.2 ± 14.4].

The mean serum 25(OH)D level was 21.23 ± 10.71 ng/mL (median: 20.70; range: 2.7–59.9). Deficiency was present in 47 patients (48.5%), insufficiency in 29 patients (29.9%), and sufficiency in 21 patients (21.6%). Overall, 76 patients (78.4%) had hypovitaminosis D.

Baseline characteristics were comparable across vitamin D categories, except for age, which increased progressively from the deficient to sufficient groups (62.2 ± 15.0, 65.3 ± 10.0, and 70.7 ± 10.8 years, *p* = 0.044). Thus, patients with adequate vitamin D status were, on average, 8.5 years older than patients with a deficiency (Table 1). Complete data for every variable analyzed was available for all 97 patients.

**Table 1:** Baseline characteristics by vitamin D status.

Characteristic	Deficient (<20 ng/mL)	Insufficient (20–29.9 ng/mL)	Sufficient (≥30 ng/mL)	*p*-value
	*n* = 47	*n* = 29	*n* = 21	
25(OH)D (ng/mL)	12.49 ± 4.64	24.44 ± 2.68	36.33 ± 7.58	<0.001
Age (years)	62.2 ± 15.0	65.3 ± 10.0	70.7 ± 10.8	0.044
Female, *n* (%)	21 (44.7)	16 (55.2)	15 (71.4)	0.121
CIRS comorbidity index	3.74 ± 1.70	4.24 ± 1.84	3.71 ± 1.55	0.415
Orthopaedic/neurological, *n*/*n*	26/21	21/8	14/7	0.300
FIM at admission	76.6 ± 13.7	79.4 ± 10.4	76.3 ± 11.2	0.571
Barthel Index at admission	31.3 ± 13.3	34.0 ± 13.6	31.9 ± 11.3	0.685
C-reactive protein (mg/L)	4.13 ± 5.18	4.80 ± 4.12	3.50 ± 3.71	0.615
Haemoglobin (g/dL)	11.40 ± 1.94	11.49 ± 2.34	11.30 ± 1.42	0.947
ICF d450 performance (0–4)	2.6 ± 1.0	2.7 ± 0.9	2.9 ± 1.0	0.678
Length of stay (days)^a^	32 [28–42]	29 [26–35]	37 [30–40]	0.170

Values are mean ± standard deviation or *n* (%), unless otherwise specified. *p*-Values from one-way ANOVA (continuous variables) and *χ*^2^ test (categorical variables).

a Median [interquartile range]; overall median 31 days [28–38], range 14–81.

CIRS, Cumulative Illness Rating Scale; FIM, Functional Independence Measure; ICF, International Classification of Functioning, Disability and Health; 25(OH)D, 25-hydroxyvitamin D.

### 3.2 Functional outcomes by vitamin D status

The FIM score increased from 77.4 ± 12.4 at admission (range 46–105) to 114.8 ± 14.5 at discharge (range 51–126), representing a mean improvement of 37.4 ± 11.8 points. The Barthel Index improved from 32.2 ± 13.1 to 89.4 ± 17.3 (mean gain: 57.2 ± 15.6). At admission, all patients were non-ambulatory (6MWT: 0 m; FAC: 0 in 73 patients, and 1–2 in the remaining 24). At discharge, the mean 6MWT distance was 230.7 ± 116.1 m, and the mean FAC was 4.0 ± 1.0. Twenty-nine patients (29.9%) achieved independent ambulation (FAC: 5). No outcome differed significantly across vitamin D categories (Table 2, Figure 2). Likewise, functional gain was unrelated to vitamin D status (ΔFIM: 37.3 ± 13.7, 36.3 ± 9.2, and 39.3 ± 9.6; *F* = 0.412, *p* = 0.663).

**Table 2:** Functional outcomes at discharge by vitamin D status.

Outcome	Deficient	Insufficient	Sufficient	*p*-value
	*n* = 47	*n* = 29	*n* = 21	
FIM at discharge	113.9 ± 16.5	115.7 ± 12.2	115.6 ± 11.8	0.838
Barthel Index at discharge	88.7 ± 19.7	89.0 ± 16.0	91.7 ± 12.3	0.803
6MWT at discharge (m)	236.1 ± 118.6	226.8 ± 126.5	224.0 ± 89.3	0.905
FAC at discharge (0–5)	4.0 ± 1.1	4.0 ± 1.0	4.1 ± 0.8	0.940
Change in FIM	37.3 ± 13.7	36.3 ± 9.2	39.3 ± 9.6	0.663
Change in Barthel Index	57.4 ± 17.1	55.0 ± 15.0	59.8 ± 12.0	0.568

Values are mean ± standard deviation; *p*-values from one-way ANOVA. FAC, Functional Ambulation Category; FIM, Functional Independence Measure; 6MWT, 6-min walk test.

**Figure 2:**
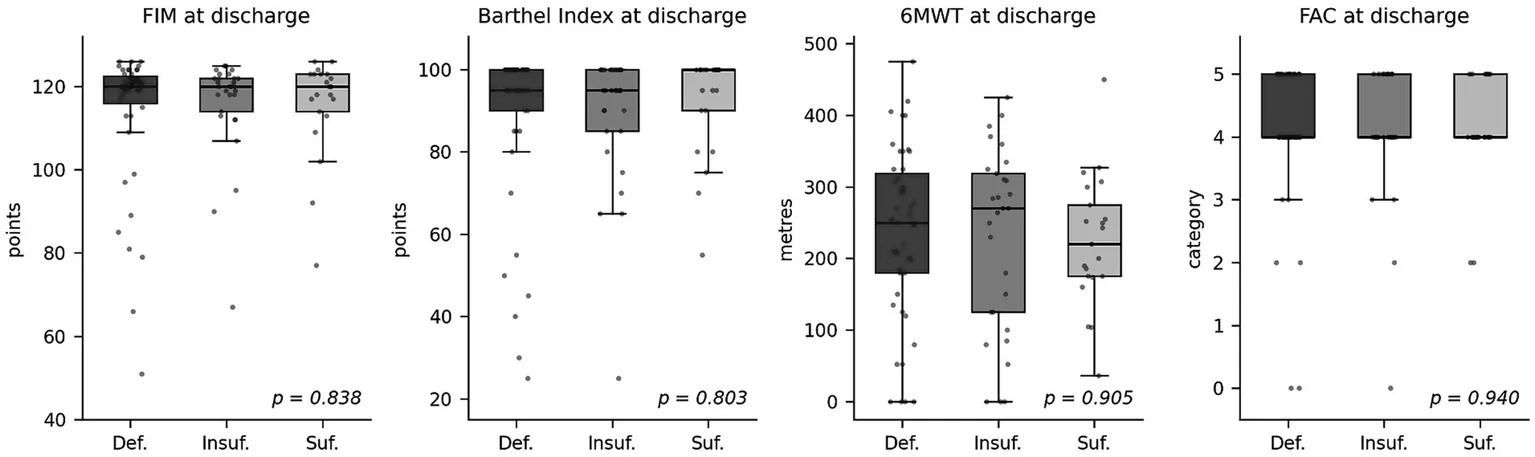
Functional outcomes at discharge by vitamin D category. Boxes show median and interquartile range, whiskers the 1.5 × IQR limits; individual patients are overlaid. *p*-Values from one-way ANOVA.

### 3.3 Univariate correlations

Serum 25(OH)D showed no significant correlation with any outcome. All coefficients were below 0.10 in absolute value, and in the rank-based analysis, they were essentially null (see Table 3).

**Table 3:** Correlations between serum 25(OH)D and functional outcomes.

Outcome	Pearson *r*	*p*-Value	Spearman *ρ*	*p*-value
FIM at discharge	+0.073	0.476	+0.001	0.993
Barthel Index at discharge	+0.057	0.579	−0.006	0.957
Change in FIM	+0.075	0.465	+0.023	0.820
Change in Barthel Index	+0.070	0.496	+0.062	0.548
6MWT at discharge	−0.066	0.518	−0.032	0.758
FAC at discharge	+0.051	0.618	+0.061	0.554
Length of stay	+0.029	0.776	+0.028	0.789

*n* = 97 for all comparisons. FAC, Functional Ambulation Category; FIM, Functional Independence Measure; 6MWT, 6-min walk test.

### 3.4 Multivariate linear regression: primary outcome

Across all three models, 25(OH)D was not associated with FIM at discharge (Model 1: *β* = 0.087, *p* = 0.425; fully adjusted Model 3: *β* = 0.095, *p* = 0.408; see Table 4). FIM at admission was the only significant predictor in every model (*β* = 0.730–0.818; *p* < 0.001). The null estimate was robust to heteroscedasticity-consistent standard errors and bootstrap resampling (95% CI: −0.123 to 0.347), and the upper confidence limit corresponds to approximately six FIM points across the full observed range of 25(OH)D—below the threshold considered clinically meaningful in inpatient rehabilitation (39, 40). When sex was added to the fully adjusted model, female patients scored approximately five points lower at discharge (*β* = −5.09, *p* = 0.037), but the 25(OH)D coefficient remained non-significant (*β* = 0.109, *p* = 0.329).

**Table 4:** Hierarchical linear regression models for FIM at discharge (*n* = 97).

Predictor	*β*	SE	*t*	95% CI	*p*-value
Model 1 (*R*^2^ = 0.396, RMSE = 11.20)					
25(OH)D (ng/mL)	+0.087	0.108	0.80	−0.128 to 0.302	0.425
FIM at admission	+0.730	0.094	7.80	0.544 to 0.916	<0.001
Model 2 (*R*^2^ = 0.415, RMSE = 11.03)					
25(OH)D (ng/mL)	+0.087	0.112	0.78	−0.136 to 0.310	0.440
FIM at admission	+0.818	0.123	6.63	0.573 to 1.063	<0.001
Age (years)	+0.092	0.098	0.94	−0.102 to 0.286	0.349
CIRS	−0.636	0.766	−0.83	−2.158 to 0.886	0.409
Neurological group	+4.475	3.122	1.43	−1.726 to 10.675	0.155
Model 3 (*R*^2^ = 0.416, RMSE = 11.01)					
25(OH)D (ng/mL)	+0.095	0.115	0.83	−0.132 to 0.323	0.408
FIM at admission	+0.820	0.125	6.58	0.572 to 1.068	<0.001
Age (years)	+0.082	0.100	0.82	−0.117 to 0.282	0.414
CIRS	−0.604	0.776	−0.78	−2.146 to 0.939	0.439
Neurological group	+5.237	3.466	1.51	−1.650 to 12.124	0.134
C-reactive protein (mg/L)	+0.134	0.299	0.45	−0.461 to 0.728	0.656
Serum calcium (mg/dL)	−0.526	2.382	−0.22	−5.259 to 4.207	0.826

CIRS, Cumulative Illness Rating Scale; FIM, Functional Independence Measure; RMSE, root mean square error; SE, standard error; 25(OH)D, 25-hydroxyvitamin D.

### 3.5 Secondary outcomes and subgroup analyses

Secondary outcomes were consistent with the primary analysis (Figure 3): 25(OH)D did not predict Barthel Index, FAC, 6MWT, or length of stay after adjustment. Comorbidity burden showed borderline associations with 6MWT (*β* = −14.56, *p* = 0.059) and FAC (*β* = −0.121, *p* = 0.060). The null finding was confirmed across all pre-specified sensitivity analyses: in patients with below-median admission FIM (*n* = 37; *r* = 0.172, *p* = 0.308); in both diagnostic subgroups (orthopedic: *r* = 0.054, *p* = 0.677; neurological: *r* = 0.025, *p* = 0.886); and with a non-significant interaction term (*β* = −0.156, *p* = 0.530). At admission, the cohort exhibited moderate-to-severe walking limitations (ICF d450 mean performance: 2.7 ± 1.0), with no differences across vitamin D categories (*r* = 0.030, *p* = 0.774). Restricting the analysis to the 64 non-supplemented patients confirmed the null result (*β* = 0.059, *p* = 0.707). Adding length of stay as a covariate did not alter the finding (*β* = 0.088, *p* = 0.437), and rehabilitation efficiency (mean 1.10 ± 0.46 FIM points/day) was unrelated to 25(OH)D (*r* = 0.050, *p* = 0.630). The categorical dummy-variable model showed no significant difference between insufficient or sufficient groups versus the deficient reference (*p* = 0.926 and *p* = 0.599), and the quadratic term was non-significant (*β* = −0.003, *p* = 0.656), excluding a threshold or nonlinear relationship.

**Figure 3:**
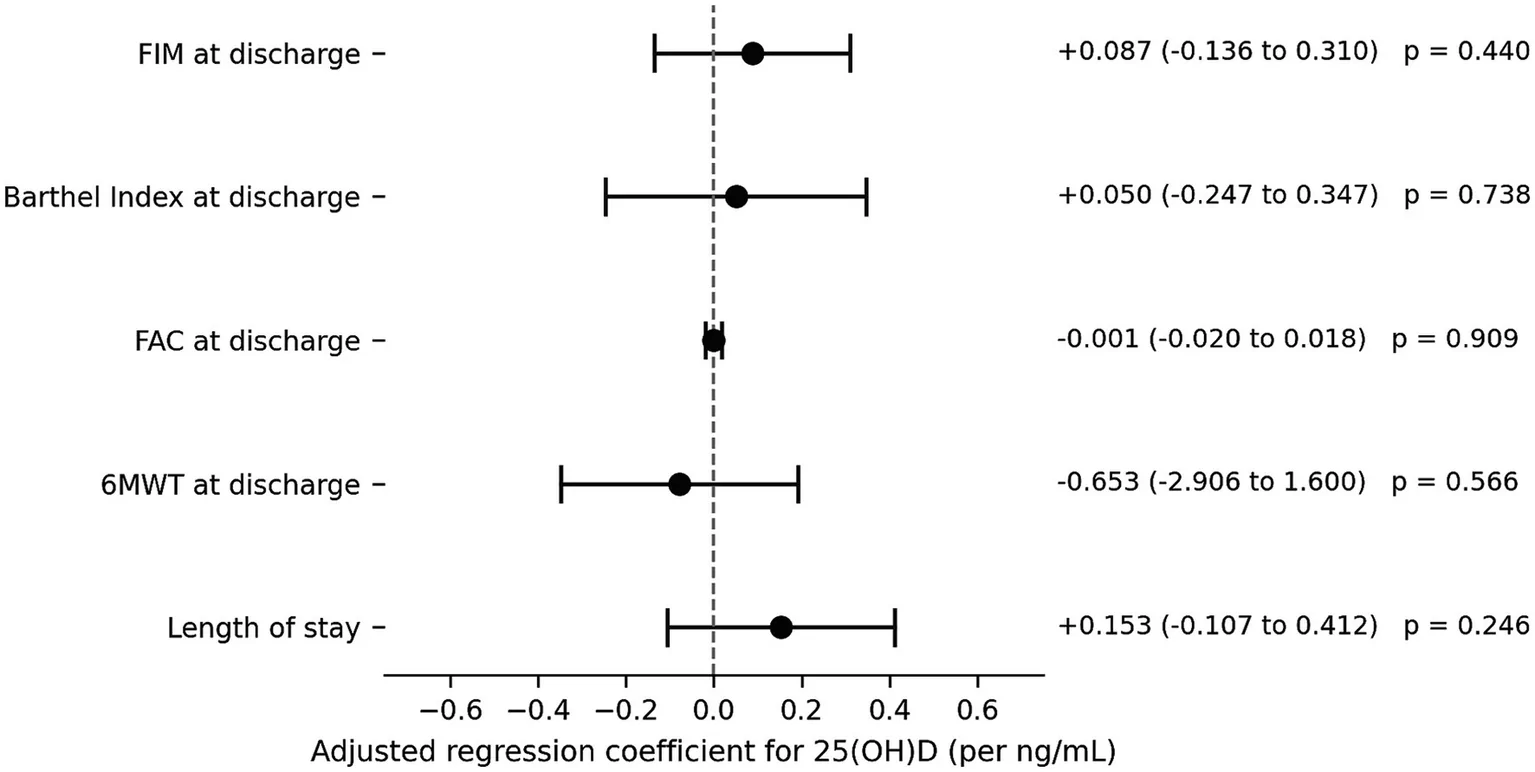
The adjusted regression coefficients for serum 25(OH)D are shown for all outcomes, along with their 95% confidence intervals. Each model is adjusted for the corresponding admission value, age, comorbidity burden, and diagnostic group (where applicable). The 6-min walk test interval is rescaled for display, and numerical values are shown on the right.

### 3.6 Vitamin D supplementation during admission

Thirty-three patients (34.0%) started cholecalciferol supplementation at the recommended dose from the Italian Society for Osteoporosis, Mineral Metabolism, and Bone Diseases (SIOMMMS) (33)during admission. The remaining 64 patients either received no supplementation or were already on treatment before admission. The supplemented patients had a significantly lower baseline 25(OH)D level (14.1 ± 9.7 versus 24.9 ± 9.3 ng/mL, *p* < 0.001), which reflects clinical selection. FIM at discharge did not differ between the two groups (113.8 ± 18.0 versus 115.3 ± 12.4, *p* = 0.626). Due to confounding by indication, this comparison is descriptive only and does not address the study objective.

## 4 Discussion

### 4.1 Summary of main findings

In this cohort of 97 patients undergoing intensive inpatient rehabilitation, baseline serum vitamin D levels were not associated with functional outcomes at discharge. This was true regardless of the measure used (FIM, Barthel Index, FAC, 6MWT, or length of stay), the statistical approach (univariate, multivariable, robust, or bootstrap), or the clinical subgroup analyzed. Hypovitaminosis D was highly prevalent (78.4%, with frank deficiency at 48.5%), yet it did not predict poorer outcomes; patients with sufficient vitamin D did not recover better.

Baseline functional status was the strongest predictor of discharge FIM, accounting for most of the explained variance (*R*^2^ ≈ 0.40). This indicates that initial disability severity, rather than serum biochemical parameters such as 25(OH)D, is the primary determinant of rehabilitation outcomes in this setting. This is consistent with our previous experience in the same setting, where functional quality outcomes after hip fracture were driven by the timing of surgery and by baseline functional status rather than by metabolic parameters (41).

Two features of the cohort strengthen this interpretation. First, patients with sufficient vitamin D were significantly older (70.7 ± 10.8 vs. 62.2 ± 15.0 years in the deficient group, *p* = 0.044). Since older age is generally associated with poorer functional outcomes, any residual confounding by age acts against the hypothesis of better recovery in the sufficient group, making the null result conservative rather than diluted. The reasons for this age distribution are not fully clear and may reflect local referral patterns.

### 4.2 Comparison with existing literature and mechanistic interpretation

Our findings align with recent negative results in related populations. Among observational studies examining baseline vitamin D as a prognostic factor, Socci et al. (42) reported no significant correlation between vitamin D and neurological outcome in traumatic brain injury, and only weak associations have been reported elsewhere (43). Randomized trials examining supplementation as a treatment have similarly yielded largely null results in post-stroke (22), severe acquired brain injury (30), and chronic spinal cord injury (29) populations, consistent with meta-analyses in community-dwelling older adults (44). These interventional findings address a different hypothesis from ours but reinforce the overall picture.

Several mechanisms may reconcile the biological plausibility of vitamin D with the absent clinical effect. Patients admitted to intensive rehabilitation represent a clinically stable, selected group, which reduces heterogeneity in recovery-driving factors and renders vitamin D less discriminatory; those with severe vitamin D-responsive conditions such as proximal myopathy would typically present as acute medical emergencies (45, 46). Vitamin D deficiency frequently co-occurs with malnutrition, immobility, and renal disease, and despite multivariable adjustment, unmeasured confounders likely remain (4, 47). A single 25(OH)D measurement within 48 h of admission may not capture longitudinal dynamics, and the acute-phase state may itself distort total 25(OH)D through reductions in vitamin D-binding protein and albumin (48, 49).

Local CYP27B1 activity in skeletal muscle and VDR polymorphisms may further dissociate serum concentration from functional response (50, 51). Finally, in a cohort with a mean 25(OH)D of 21 ng/mL and few patients below 10 ng/mL, the difference between deficient and sufficient levels may simply be insufficient to produce measurable functional differences over a median 31-day stay—a limitation quantified by our confidence intervals, which exclude an effect larger than approximately 0.32 FIM points per ng/mL (45, 52, 53).

### 4.3 The role of ceiling effects

The distribution of outcomes is particularly important because it defines the conditions under which our null result holds. In this cohort, recovery on the ordinal scales was nearly complete: 44.3% of patients achieved the maximum Barthel score, and the FIM distribution at discharge was strongly left-skewed, with an average score of 114.8 out of 126. In a population that almost fully recovers within the rehabilitation stay, there is limited residual variance for any prognostic factor to explain. This constrains the discriminatory power of the scales rather than the biological plausibility of the hypothesis. The 6MWT, which is continuous and free from a ceiling effect in this range, exhibited the greatest residual variability. However, the adjusted model explained only 5.7% of this variability, indicating that determinants other than nutritional status dominate walking recovery. Outcome measures that are more sensitive to nutritional effects, such as grip strength, muscle ultrasound, and bioelectrical impedance, would be better suited to detecting small effects.

### 4.4 Implications for clinical practice and research

Our data do not support using baseline serum vitamin D levels to stratify prognosis in intensive inpatient rehabilitation. Clinicians should not adjust prognosis based on admission vitamin D levels. However, this does not diminish the importance of correcting hypovitaminosis D, which remains justified for the prevention of osteoporosis and fractures, for immune competence and for general nutritional optimization, and because adequate vitamin D status enhances the densitometric response to antiresorptive treatment (54). The absence of a demonstrable effect on short-term functional recovery does not negate the benefits of long-term skeletal health supplementation, particularly in a cohort where nearly four in five patients had levels below 30 ng/mL.

Future studies should employ randomized designs with structured, time-limited supplementation protocols and focus on populations with severe deficiency (below 10–15 ng/mL) to determine if a threshold exists below which vitamin D becomes limiting. These studies should also investigate mechanistic endpoints, such as inflammatory cytokines and muscle protein synthesis, rather than functional scales alone. They should use dynamic biomarkers and repeated measurements rather than a single admission value, adopt outcome measures less affected by ceiling effects, and examine high-risk populations, such as those with post-critical illness or early post-stroke, in whom an effect might be more readily detectable.

### 4.5 Strengths and limitations

The strengths of this study include the systematic and standardized assessment of functional outcomes at admission and discharge as part of routine intensive rehabilitation practice, which yielded a dataset complete for every variable analyzed in the final cohort; the use of multiple outcome measures spanning independence, ambulation and walking capacity, which reduces the risk of selective reporting; comprehensive multivariable adjustment with robust and bootstrap-based confirmation of the main estimate; prespecified subgroup and interaction analyses; and transparent reporting of null results with confidence intervals and an explicit statement of statistical power.

Several limitations should be acknowledged. The observational design cannot exclude residual confounding, and vitamin D was measured once, within 48 h of admission, so that longitudinal dynamics were not captured; the acute-phase state at the time of sampling may itself have distorted total 25(OH)D. Several potential confounders were unavailable for the full cohort, including nutritional status (albumin available in fewer than 10% of patients), and season of admission; previous vitamin D supplementation is addressed in the sensitivity analysis below. The study was conducted over a period of 21 months, during which time there may have been changes in vitamin D status due to seasonal variations in 25(OH)D levels. The time interval between acute event and rehabilitation admission was not systematically recorded and could not be analyzed as a potential modifier of the vitamin D–outcome relationship, particularly in stroke patients.

In-hospital vitamin D supplementation, initiated according to standard clinical protocol in deficient patients, represents a potential confounder; however, a sensitivity analysis restricted to the 64 non-supplemented patients yielded results fully consistent with the primary analysis (*β* = 0.059, *p* = 0.707).

The cohort was only moderately deficient on average (21.23 ng/mL), with few patients below 10 ng/mL, limiting generalizability to severely deficient populations. Subgroup sizes were modest (neurological *n* = 36), and the study was powered to detect moderate but not small effects: the confidence interval for the primary estimate excludes an effect larger than about 0.32 FIM points per ng/mL but remains compatible with a negligible one. Ceiling effects in the ordinal scales reduced outcome sensitivity, as discussed above. The wide variation in length of stay (range 14–81 days) means that functional outcomes were assessed after different durations of rehabilitation; however, sensitivity analyses adjusting for length of stay and rehabilitation efficiency analyses confirmed the null finding.

The composition of the total FIM score differs between the two diagnostic groups, since the cognitive subscale is near-ceiling among orthopedic patients by selection; three cognition-free outcomes yielded the same null result, the total FIM and the Barthel Index at discharge were closely correlated (*r* = 0.96), and no interaction with diagnostic group was detected, making this an unlikely explanation for the absent association. ICF qualifiers were available at admission only and could therefore be used as baseline descriptors rather than outcomes. No direct measure of muscle mass or sarcopenia status was available, although appendicular skeletal muscle mass can be estimated from a small number of anthropometric variables at no additional cost (55), and its inclusion would have allowed the hypothesized muscular pathway to be tested more directly. Requiring complete assessments at both time points may have introduced selection bias by excluding patients who deteriorated or were transferred before discharge, which could have enriched the cohort with more stable cases. Additionally, both diagnostic groups were internally heterogeneous—the neurological group comprised primarily post-stroke patients, as well as cases of Guillain-Barré syndrome and post-neurosurgical cases—which may have reduced the ability to detect subgroup-specific effects. Finally, the study was conducted at a single center and could not assess outcomes beyond discharge.

## 5 Conclusion

In conclusion, baseline serum 25(OH)D did not predict functional recovery in intensive inpatient rehabilitation despite the high prevalence of hypovitaminosis D in this cohort. This finding was consistent across five outcome measures, three regression models, and both robust and bootstrap estimations, and across every subgroup examined. These results do not negate the biological importance of vitamin D in the musculoskeletal and neurological systems, but suggest that a single admission measurement of total 25(OH)D is not a clinically useful prognostic factor for this population. Randomized trials with structured supplementation and mechanistic endpoints are needed to determine whether intervention, as opposed to baseline status, can improve rehabilitation outcomes.

## Data Availability

The original contributions presented in the study are included in the article/supplementary material, further inquiries can be directed to the corresponding author.
